# Editorial: Clinical and Molecular Epidemiology of Thyroid Cancer of Follicular Origin

**DOI:** 10.3389/fendo.2018.00067

**Published:** 2018-03-12

**Authors:** Roberta Malaguarnera, Veronica Vella, Gabriella Pellegriti, Antonino Belfiore

**Affiliations:** ^1^Endocrinology, Department of Health Sciences, University Magna Graecia of Catanzaro, Catanzaro, Italy; ^2^Endocrinology, Department of Clinical and Experimental Medicine, University of Catania, Garibaldi-Nesima Hospital, Catania, Italy; ^3^School of Human and Social Sciences, Kore University of Enna, Enna, Italy

**Keywords:** thyroid cancer, epidemiology, environmental endocrine disruptors, life style changes, hyperinsulinemia, thyroid autoimmunity, molecular profiling, multikinase inhibitors

**Editorial on the Research Topic**

**Clinical and Molecular Epidemiology of Thyroid Cancer of Follicular Origin**

Thyroid cancer (TC) accounts for 3% of all human cancers and includes well-differentiated thyroid carcinomas (DTC) (papillary and follicular) that represent the most frequent subtypes (85–90% of total), but also aggressive tumors such as poorly differentiated thyroid cancers (PDTC) (2–10% of total) and anaplastic thyroid carcinomas (ATC) (<1% of total). While DTCs can be cured with surgery and radioiodine, PDTCs and ATCs are resistant to both radioiodine and chemotherapy (1, 2).

Importantly, the incidence of TC has increased worldwide over the past 20 years, and the epidemiologic landscape of TC is also considerably changing in view of a predominant increase of the papillary histotype (PTC) (3).

Mutational and non-mutational events in survival mediators (i.e., MAPK, PI3K, mTOR) and/or tyrosine kinase receptors (i.e., IGF-IR, insulin receptor, EGFR, HGF/SF receptor) as well as epigenetic modifications, including DNA methylation and non-coding RNA mediated events, have been found to be associated with TC initiation and progression (4–6). However, the risk factors underlying specific molecular alterations are poorly understood, and the factors involved in the changing epidemiology of TC are mostly unknown.

This Research Topic includes three original articles, three mini reviews, and one review focusing on the molecular and clinical aspects of TC epidemiology.

Age, ethnicity, genetic predisposition, and sex are well-recognized, non-modifiable risk factors for TC (3). DTC occurs more frequently in females (female-to-male ratio of almost 3:1 across different countries and ethnicities) with an incidence peak during the reproductive period (7). The minireview of Moleti et al. summarizes clinical and experimental studies indicating a putative role of estrogens and cognate receptors in TC growth. The authors also focus on the possible role of placental hormones and other pregnancy-related growth factors in the occurrence and worse outcome of DTCs diagnosed in women during gestation.

In addition, epidemiological studies suggest that changing environmental factors and casual genetic aberrations more frequent in a background of increased cell replication may play a role in the increased TC incidence (8). A register-based epidemiological survey performed in Sicily has shown that the incidence of DTC (mostly PTC) is higher in the volcanic area of eastern Sicily around the Mt. Etna as compared to the non-volcanic areas (Western of Sicily). Tavarelli et al. show that, in the volcanic area of Sicily, the incidence not only of DTC but also of ATC is increased, possibly in relation to volcano-derived contamination of the atmosphere, soil, and water with endocrine disrupting chemicals. These data suggest that environmental factors may influence ATC incidence indirectly by promoting DTCs, which, in turn, may progress to ATCs after de-differentiation.

Beyond environmental carcinogens, other modifiable risk factors, such as those related to lifestyle, are suspected to play a role in the changing epidemiology of TC. Malaguarnera et al. reviewed pieces of evidence suggesting that the recent epidemic of metabolic disorders associated with insulin resistance, such as obesity, type 2 diabetes mellitus, and others, may be causally linked to the rising TC risk, either directly through hyperinsulinemia or by potentiating the effects of other TC risk factors including iodine-deficiency, thyroid stimulating hormone and estrogen signaling, thyroid angiogenesis, thyroid autoimmunity, and others. These studies may have important implications in terms of TC prevention and treatment, as insulin-resistance and related disorders are responsive to lifestyle changes and/or to insulin-sensitizing drugs, which may be considered as preventive measures.

The article by Ferrari et al. reports that the modification of other eco-factors, such as iodine and selenium intake, vitamin D deficiency, intestinal dysbiosis, exposure to radiation, viruses, and chemicals, may affect chronic autoimmune thyroiditis (AIT), which may be associated with TC risk. In this context, the impact of the most common AIT, the chronic lymphocytic thyroiditis (CLT), on TC onset and outcome is still controversial. Some papers have reported an increased risk of PTC in patients with CLT (9). A number of studies observed that PTC patients with CLT have a better prognosis (10, 11). The original article of Ieni et al. found that CLT was present in 33.3% of PTCs, and was associated with a more favorable TNM staging. This frequency of CLT in PTCs is roughly double to that reported in a recent meta-analysis (18.9%) (9) and suggests possible significant differences in the diagnostic criteria of CLT and/or different occurrence rates associated to the geographical area. Overall, these data confirm that the frequency and significance of concomitant AIT in PTCs are still debated.

Some studies have emphasized that the increasing TC incidence could be the result of an early diagnosis of PTC microcarcinomas (12). However, other studies have suggested that TC incidence is truly increasing, likely because some rising risk factors might favor certain common molecular aberrations found in PTCs (13). In light of these considerations, molecular profiling of thyroid nodules, especially those with indeterminate cytology, could increase preoperative diagnostic accuracy. The study of Censi et al. investigated the frequency and significance of RAS, TERT promoter, and BRAF gene mutations in patients undergoing thyroid surgery for nodules with indeterminate cytology. They found that the use of combined molecular tests increases the likelihood for cancer risk from 27 to 62%, allowing identifying 48% of malignant lesions in this group of indeterminate lesions. However, the sensitivity of TERT promoter mutation testing alone for predicting cancer risk was low and not cost-effective. Combined mutational analysis of driver genetic alterations may also be appropriate for all forms of DTC, PDTC, and ATC in order to provide a personalized approach in terms of diagnosis, prognosis, and therapy.

As highlighted in the paper of Tumino et al., the classical TC management, including surgery, TSH suppression, and radioiodine therapy, remains effective for patients at high risk of recurrence and mortality. However, new target therapies, especially based on multikinase inhibitors, have become available and have opened new perspectives for the therapy of iodine refractory and advanced TCs with various histotypes.

## Author Contributions

All authors listed have made a substantial, direct, and intellectual contribution to the work and approved it for publication.

## Conflict of Interest Statement

The authors declare that the research was conducted in the absence of any commercial or financial relationships that could be construed as a potential conflict of interest. The reviewer GC and handling Editor declared their shared affiliation.
